# The Persian internet gaming cognition scale: A psychometric evaluation among Iranian adolescents

**DOI:** 10.1016/j.abrep.2026.100745

**Published:** 2026-08-28

**Authors:** Fereshteh Rajaeiramsheh, Hojjatollah Farahani, Ahmad Ashouri, Komeil Zahedi Tajrishi

**Affiliations:** a Faculty of Behavioral Sciences and Mental Health, Tehran Institute of Psychiatry, Iran University of Medical Sciences, Tehran, Iran.; b Department of Psychology, Tarbiat Modares University, Tehran, Iran.

**Keywords:** Internet gaming cognition scale, Gaming cognitions, Problematic gaming, Adolescents, Psychometrics, Item response theory

## Abstract

**Background:**

Maladaptive gaming-related cognitions are theorized to maintain problematic gaming in adolescence. The Internet Gaming Cognition Scale (IGCS) assesses these cognitions, but no culturally adapted Persian version had been evaluated for Iranian adolescents.

**Methods:**

After permission from the original author, the IGCS was translated and culturally adapted using forward translation, reconciliation, back-translation, and author confirmation. Online data were collected from 1191 Iranian adolescents. Construct validity was examined using confirmatory factor analysis (CFA) with DWLS estimation. Reliability, item-total statistics, external validity, AVE, HTMT, and multidimensional graded response model (GRM) parameters were evaluated.

**Results:**

The four-factor CFA model showed acceptable-to-good fit (CFI = 0.990, TLI = 0.989, RMSEA = 0.079, SRMR = 0.060). Total-scale reliability was excellent (α = 0.944), and corrected item-total correlations ranged from 0.226 to 0.732. AVE values ranged from 0.460 to 0.618, whereas HTMT indices indicated high factor overlap. The multidimensional GRM fit better than the unidimensional model, χ^2^(6) = 212.090, *p* < .001. Non-zero discrimination parameters ranged from 0.437 to 3.765, and ordered thresholds supported the five response categories. The IGCS correlated positively with gaming addiction (*r* = 0.660, *p* < .001) and negatively with self-esteem (*r* = −0.237, *p* < .001).

**Conclusions:**

The Persian IGCS showed satisfactory structural fit, excellent reliability, and acceptable item-level evidence. Given the high overlap among factors, the total score is recommended as the primary indicator, whereas subscale scores should be interpreted cautiously.

## Highlights

• The Persian IGCS was evaluated in 1191 predominantly adolescent participants.
• Four-factor models were relatively superior, but the domains overlapped strongly.
• E1 and E2 showed weak discrimination; E19 and E24 were locally dependent.
• IGCS scores related to gaming problems and inversely to self-esteem.

## 1 Introduction

Digital gaming is a routine part of adolescence and can serve recreational, social, and cognitive functions. For some young people, however, gaming becomes difficult to control, takes priority over other activities, and continues despite harmful consequences, leading to clinically meaningful impairment. This pattern is recognized as gaming disorder in the ICD-11, whereas Internet gaming disorder remains a condition for further study in the DSM-5 (American Psychiatric Association, 2013; World Health Organization, 2020). Prevalence estimates differ across instruments and populations, but problematic gaming affects a meaningful minority of adolescents and is reported more often among boys (Gao et al., 2022; Stevens et al., 2021). Identifying the psychological processes associated with persistent difficulties is therefore an important complement to symptom-based assessment.

Cognitive-behavioral accounts propose that the meaning young people attach to gaming experiences can help sustain problematic play. King and Delfabbro's (2014) model distinguishes four related belief domains: overvaluation of gaming rewards, maladaptive and inflexible rules about play, gaming-based self-esteem, and gaming as a route to social acceptance. These beliefs may increase the perceived value of gaming, strengthen urges to continue or resume play, and make gaming-related goals feel disproportionately important relative to competing activities. The Interaction of Person-Affect-Cognition-Execution model similarly places gaming-specific expectancies within the interplay of personal vulnerabilities, affective responses, and executive-control processes (Brand et al., 2019).

Empirical findings are consistent with this cognitive account across different age groups and study designs. Gaming-related cognitions have been associated with problematic gaming in adolescent and adult samples, and longitudinal evidence suggests that maladaptive cognitions can predict subsequent changes in gaming problems (Forrest et al., 2017; Li & Wang, 2013; Moudiab & Spada, 2019). Recurrent thoughts about gaming, unfinished goals, anticipated rewards, and beliefs concerning competence or social recognition may therefore represent maintaining cognitive processes rather than merely accompanying symptoms. This distinction is clinically and theoretically important because cognitive processes may provide targets for assessment and intervention even when symptom severity is measured separately.

Most widely used gaming measures are symptom-focused, including the Internet Gaming Disorder Scale-Short Form and the Gaming Disorder Test (Maldonado-Murciano et al., 2024). Persian measures of gaming symptoms are also available for Iranian adolescents and young people (Abdoli et al., 2021; Lin et al., 2019). Measures of cognitive processes, however, address related but non-equivalent constructs. The Metacognitions About Online Gaming Scale assesses beliefs about gaming as a way to regulate internal experiences and beliefs about uncontrollability and harm, and a Persian version has been evaluated in Iranian adolescents (Akbari et al., 2021; Lin et al., 2024). The Time Perspective Scale of Perceived Benefits and Harms of Internet Gaming captures immediate and future-oriented appraisals of gaming consequences (Li et al., 2025). None of these measures directly assesses the four content-specific belief domains represented in the IGCS. The IGCS is therefore particularly relevant when the research question concerns what adolescents believe about gaming rather than how many diagnostic symptoms they report.

The original 24-item IGCS was examined in 824 adolescents and showed good subscale reliability (α = 0.81–0.90), with higher scores among adolescents with Internet gaming disorder than among highly engaged peers without the disorder (King & Delfabbro, 2016). Cross-cultural findings, however, have not been uniform. The original four-factor structure was not retained in 755 Chinese adolescents, leading to a 15-item, three-factor revision (Yu et al., 2020), whereas a Turkish adaptation in a mixed adolescent-adult sample retained the original multidimensional specification (Cakiroglu & Gormez, 2020). Thus, even studies conducted partly or entirely in adolescent samples have not reproduced an identical measurement structure.

These differences may reflect translation, item selection, cultural meanings, sample composition, and developmental context. During adolescence, beliefs concerning reward, competence, self-worth, and recognition from peers may be particularly relevant to the meaning attached to gaming. At the same time, the inconsistent structures reported across adolescent samples suggest that developmental stage alone is unlikely to explain cross-cultural differences. Instead, age and cultural context may interact with the way particular gaming-related beliefs are interpreted and endorsed. Evaluating the Persian IGCS specifically in an adolescent-focused sample is therefore important not only as a linguistic adaptation, but also as a test of whether the theoretically proposed domains remain distinguishable in a different developmental and cultural setting.

A related scoring question is whether the IGCS is best interpreted through one total score, four domain scores, or both. Strong factor correlations alone neither establish unidimensionality nor invalidate the subscales; comparative model fit, reliability, factor distinctiveness, and item functioning must be considered together. General self-esteem refers to an individual's overall evaluation of personal worth and self-value. It is conceptually distinct from gaming-specific cognitions, but it is relevant to the cognitive domains assessed by the IGCS because gaming may become a context in which some individuals seek competence, achievement, or social recognition that contributes to their sense of self-worth. Consistent with this distinction, lower general self-esteem has shown a reliable but typically modest association with gaming disorder (Kavanagh et al., 2023). Self-esteem was therefore included as a theoretically related but non-equivalent external construct: we expected IGCS scores to correlate inversely with self-esteem, but more weakly than with gaming-problem severity.

The present study translated and culturally adapted the IGCS and evaluated its structure, reliability, external associations, and item functioning in an Iranian adolescent-focused sample. We expected the correlated four-factor model to provide an acceptable representation of the data, the total and domain scores to show adequate internal consistency, and IGCS scores to correlate positively with gaming-problem severity and more modestly and inversely with self-esteem. At the item level, we expected a correlated four-factor graded response model to outperform a unidimensional model. Supplementary analyses examined factor distinctiveness, bifactor and second-order structures, gender invariance, Mokken scalability, monotonicity, local dependence, and exploratory item-reduced specifications.

## 2 Methods

### 2.1 Participants and procedure

This cross-sectional psychometric study included 1191 respondents (M age = 15.55 years, SD = 1.87; observed range = 10–37 years), comprising 633 girls (53.1%) and 558 boys (46.9%). Recruitment targeted adolescents aged 12–18 years through online networks and study invitations distributed with assistance from education authorities in several cities. Most respondents (*n* = 1170; 98.2%) reported ages within the intended 12–18-year range. Fourteen respondents reported ages below 12 years and seven reported ages above 18 years. These 21 records (1.8%) were retained in the primary analyses; to evaluate whether their inclusion materially influenced the findings, additional sensitivity analyses were conducted using only respondents aged 12–18 years (*n* = 1170). Participants reported an average of 2.20 h of gaming per day (SD = 2.66). Further characteristics are shown in Table 1.

**Table 1:** Participant characteristics (N = 1191).

Characteristic	Value
Age, M (SD), years	15.55 (1.87)
Aged 12–18 years, n (%)	1170 (98.2)
Girls, n (%)	633 (53.1)
Boys, n (%)	558 (46.9)
Daily gaming time, M (SD), hours	2.20 (2.66)
<1 h/day, n (%)	362 (30.4)
1–3 h/day, n (%)	595 (50.0)
>3 h/day, n (%)	234 (19.6)
Low socioeconomic status, n (%)	61 (5.1)
Middle socioeconomic status, n (%)	497 (41.7)
High socioeconomic status, n (%)	633 (53.1)

*Note.* Fourteen respondents reported ages below 12 years and seven reported ages above 18 years; these 21 respondents (1.8%) were retained in the primary analyses. Age-restricted score-level sensitivity analyses are reported in the text and Supplementary Table S13.

The Ethics Committee of Iran University of Medical Sciences approved the study (IR.IUMS.REC.1404.06). Participation was voluntary. Electronic informed consent was obtained from a parent or legal guardian for every participant. Respondents younger than 18 years additionally provided assent, whereas those reporting age 18 or older provided their own informed consent in addition to parental consent. All questionnaire responses were required before online submission; therefore, the analytic dataset contained no item-level missing data.

### 2.2 Measures

#### 2.2.1 Internet gaming cognition scale

The IGCS is a 24-item self-report measure of maladaptive gaming-related beliefs (King & Delfabbro, 2016). It comprises overvaluation of gaming rewards (5 items; e.g., viewing in-game rewards as highly important), maladaptive and inflexible rules about gaming (8 items; e.g., feeling compelled to complete a gaming goal immediately), gaming-based self-esteem (6 items; e.g., deriving self-worth from gaming achievements), and gaming as a means of social acceptance (5 items; e.g., seeking recognition from other players). Responses range from 0 (never) to 4 (always), yielding a total score from 0 to 96; higher scores indicate stronger endorsement of maladaptive gaming-related cognitions. With permission from the scale author, two bilingual team members independently translated the items into Persian and reconciled their versions. An independent bilingual translator, blinded to the original wording, then completed the back-translation. The research team, subject-matter experts, and the original author reviewed conceptual and linguistic discrepancies, and adolescents pilot-tested the provisional version for clarity. The final version retained the original 24 items, four domains, response options, and scoring procedure. The complete English and Persian item sets are provided in Supplementary Table S1.

#### 2.2.2 Computer game addiction questionnaire

The 20-item Computer Game Addiction Questionnaire, based on Whang and Chang's (2002) measure, was used as a continuous indicator of gaming problems. Items are rated from 1 to 5 and summed (20−100), with higher scores indicating greater problems. A Persian version had previously been used with Iranian adolescents (Abedini et al., 2012), and internal consistency was high in this sample (α = 0.93). The measure was selected because it was embedded in the parent study, had prior Iranian adolescent use, and provided a continuous external correlate. Because it predates current DSM-5 and ICD-11 formulations and is not diagnostic, it was not used to establish gaming disorder, diagnostic accuracy, or criterion validity.

#### 2.2.3 Eysenck-Wilson self-esteem scale

General self-esteem was assessed with the 30-item Eysenck-Wilson Self-Esteem Scale (Eysenck & Wilson, 1975), which has also been used in Iranian research (Hormozi Nezhad et al., 2000). The Persian-language form used in the parent study followed the instrument's keyed scoring procedure: responses in the keyed direction received 1, responses in the opposite direction received 0, and uncertain responses received 0.5. Scores range from 0 to 30, with higher scores indicating greater self-esteem. Internal consistency was good in the present sample (α = 0.88).

### 2.3 Data analysis

Analyses were conducted in R 4.5.3. IGCS responses were retained in their original 0–4 coding, with 0 treated as the valid never category. Item distributions and endpoint frequencies were summarized; potential floor or ceiling effects were defined as more than 15% selecting the lowest or highest category.

The prespecified correlated four-factor CFA was estimated using diagonally weighted least squares. Fit was evaluated jointly using CFI, TLI, RMSEA with 90% confidence intervals, and SRMR. Bifactor and second-order models examined whether shared covariance could be represented by a general construct. A post hoc 22-item model excluding E1 and E2 evaluated sensitivity to the two weakest items. Internal consistency was estimated using Cronbach's alpha for total and domain scores, ordinal alpha, and McDonald's omega. Corrected item-total and item-subscale correlations, alpha if deleted, average variance extracted (AVE), maximum and average shared variance, and heterotrait-monotrait ratios (HTMT) were examined.

Gender measurement invariance was evaluated through configural, metric, and scalar models for ordered indicators. Changes no greater than 0.010 in CFI and 0.015 in RMSEA were considered approximate evidence of invariance, provided the less constrained model had adequate fit (Chen, 2007). Latent means were not interpreted unless scalar invariance was supported.

A correlated four-factor multidimensional graded response model (GRM) was compared with a unidimensional GRM using likelihood-ratio testing and information criteria. Absolute fit was assessed with M2-based CFI, TLI, RMSEA, and SRMSR. Item discrimination, four ordered thresholds, test information, and empirical reliability were estimated. Mokken H and response-function plots supplemented assessment of scalability and monotonicity. Yen's Q3 residual correlations assessed local dependence. Exploratory models removed E19 or E24, the strongest local-dependence pair; these and the 22-item analysis were not treated as validation of a shortened scale. Pearson correlations examined prespecified external associations with gaming problems and self-esteem. Two-sided α was 0.05.

As an age-range sensitivity check, internal-consistency estimates, external correlations, and the inter-item correlation pattern were recomputed after restricting the sample to respondents aged 12–18 years (*n* = 1170).

## 3 Results

### 3.1 Item distributions

All five response categories were used for every IGCS item. Item means ranged from 0.92 to 2.13, and skewness from −0.14 to 1.17. More than 15% selected the lowest category for every item, most notably E21 (55.75%); seven items also exceeded the 15% upper-endpoint criterion. Item-level results are in Supplementary Table S2.

### 3.2 Factor structure and reliability

**Fig. 1:**
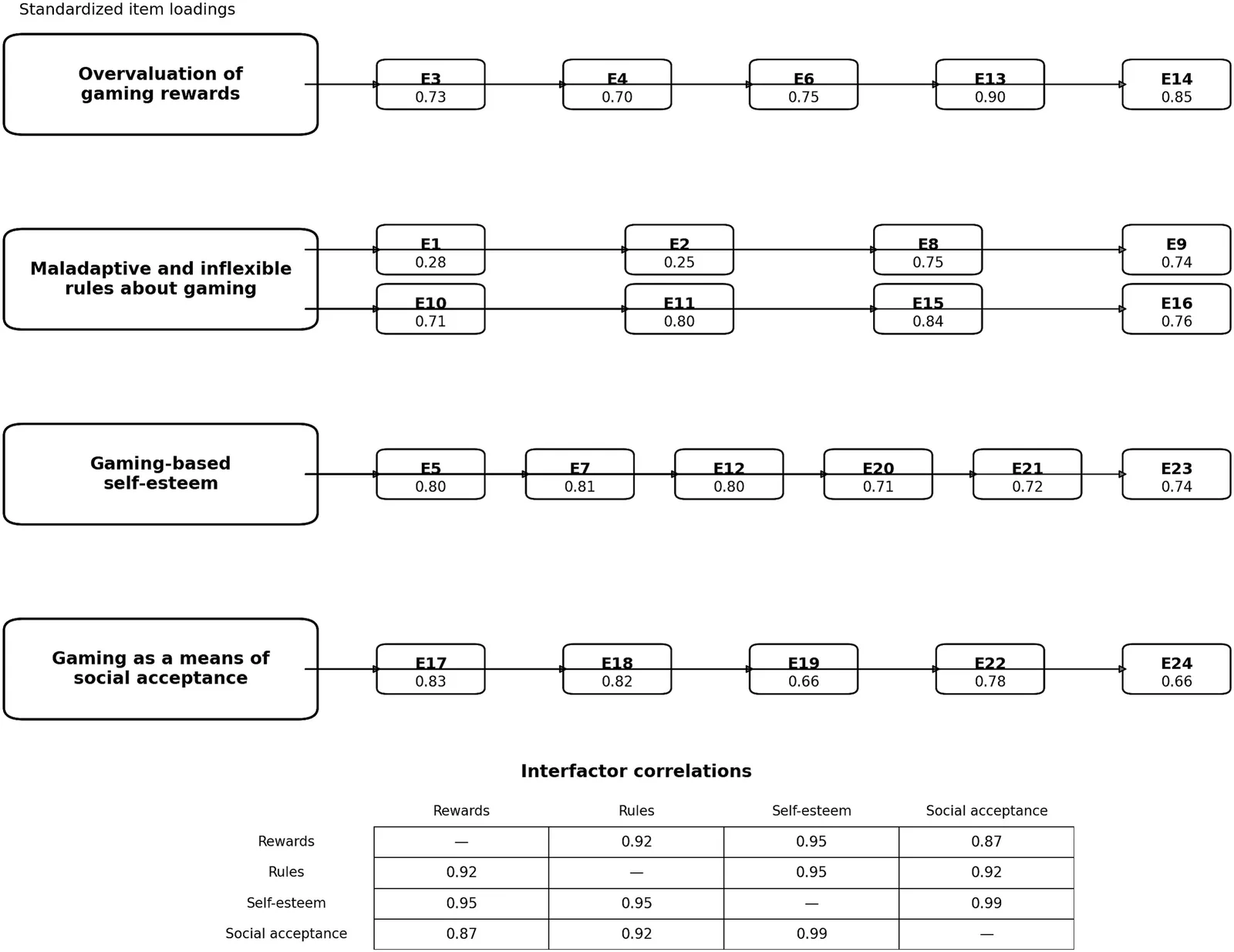
Standardized loadings and interfactor correlations for the correlated four-factor CFA model. Note. Item labels correspond to the original 24-item IGCS. Full estimates are provided in Supplementary Table S3.

The correlated four-factor CFA showed strong incremental but less favorable absolute fit, χ^2^(245) = 2064.57, *p* < .001, CFI = 0.99, TLI = 0.99, RMSEA = 0.08, 90% CI [0.076, 0.082], and SRMR = 0.06. Loadings ranged from 0.25 to 0.90. E1 (0.28) and E2 (0.25) were substantially weaker than the remaining items (0.66–0.90), and factor correlations were high (Fig. 1). The bifactor model was inadmissible. The second-order model required a residual constraint, contained a near-unit higher-order loading, and fit significantly worse than the correlated model, Δχ^2^(2) = 40.86, *p* < .001. The 22-item sensitivity model modestly improved CFI, TLI, and SRMR, but RMSEA was unchanged and factor correlations remained 0.92–0.96 (Table 2).

**Table 2:** Structural model and gender-invariance results.

Panel A. Confirmatory factor-analysis models.					
Model	χ^2^ (df)	CFI	TLI	RMSEA [90% CI]	**SRMR**
Correlated four-factor	2064.57 (245)	0.990	0.989	0.079 [0.076, 0.082]	0.060
Second-order	2105.43 (247)	0.990	0.989	0.080	0.061
Bifactor	No admissible solution	–	–	–	–
22-item sensitivity	1706.32 (202)	0.992	0.991	0.079 [0.076, 0.083]	0.056


Panel B. Gender measurement invariance						
Model	χ^2^ (df)	CFI	TLI	RMSEA	SRMR	Conclusion
Configural	4883.76 (492)	0.896	0.883	0.123	0.084	Inadequate baseline fit
Metric	3735.56 (512)	0.924	0.918	0.103	0.088	Not supported
Scalar	4631.89 (560)	0.903	0.905	0.111	0.084	Not supported

*Note.* The second-order model fit worse than the correlated model, Δχ^2^(2) = 40.86, *p* < .001. The 22-item model excluded E1 and E2. Gender latent means were not interpreted because configural fit was inadequate.

Total-score reliability was high (α = 0.94). Domain alphas ranged from 0.81 to 0.85, ordinal alphas from 0.84 to 0.89, and omegas from 0.79 to 0.86. E1 and E2 had the weakest corrected item-total and item-domain correlations. AVE exceeded 0.50 for three domains but not the rules domain (0.46). AVE was lower than shared variance for every factor, and HTMT ranged from 0.89 to 1.00, indicating limited factor distinctiveness (Table 3; Supplementary Tables S3–S6).

**Table 3:** Reliability, item-response, and external-association summary.

Panel A. Reliability and construct-level indices.								
Score	Items	Loading range	α	Ordinal α	ω	Corrected r	AVE	Max HTMT
Total	24	–	0.944	–	–	0.226–0.732	–	–
Rewards	5	0.695–0.896	0.806	0.855	0.854	0.482–0.682	0.618	0.999
Rules	8	0.254–0.841	0.806	0.843	0.832	0.237–0.667	0.460	0.974
Gaming self-esteem	6	0.711–0.806	0.850	0.889	0.864	0.561–0.720	0.583	0.999
Social acceptance	5	0.659–0.826	0.844	0.881	0.792	0.555–0.702	0.564	0.947


Panel B. Multidimensional graded response model				
Domain	a range	Threshold range	H (SE)	EAP reliability
Rewards	1.438–3.765	−1.259–1.780	0.485 (0.017)	0.934
Rules	0.437–3.169	−3.603–4.017	0.363 (0.014)	0.937
Gaming self-esteem	1.954–2.685	−0.977–2.058	0.527 (0.015)	0.941
Social acceptance	2.052–2.790	−0.690–1.774	0.546 (0.016)	0.918


Panel C. External associations		
IGCS score	Gaming problems, r	Self-esteem, r
Total	0.660	−0.237
Rewards	0.586	−0.226
Rules	0.610	−0.246
Gaming self-esteem	0.644	−0.232
Social acceptance	0.559	−0.149

*Note.* All external correlations were *p* < .001. Detailed item-level and model results are provided in the Supplementary Material.

### 3.3 Gender measurement invariance

The configural model had inadequate fit, χ^2^(492) = 4883.76, CFI = 0.90, TLI = 0.88, RMSEA = 0.12, SRMR = 0.08. Metric and scalar models also remained inadequate. Because a comparable baseline structure was not established, gender invariance was not supported and latent means were not interpreted (Table 2; Supplementary Table S7).

### 3.4 Graded response model and item diagnostics

**Fig. 2:**
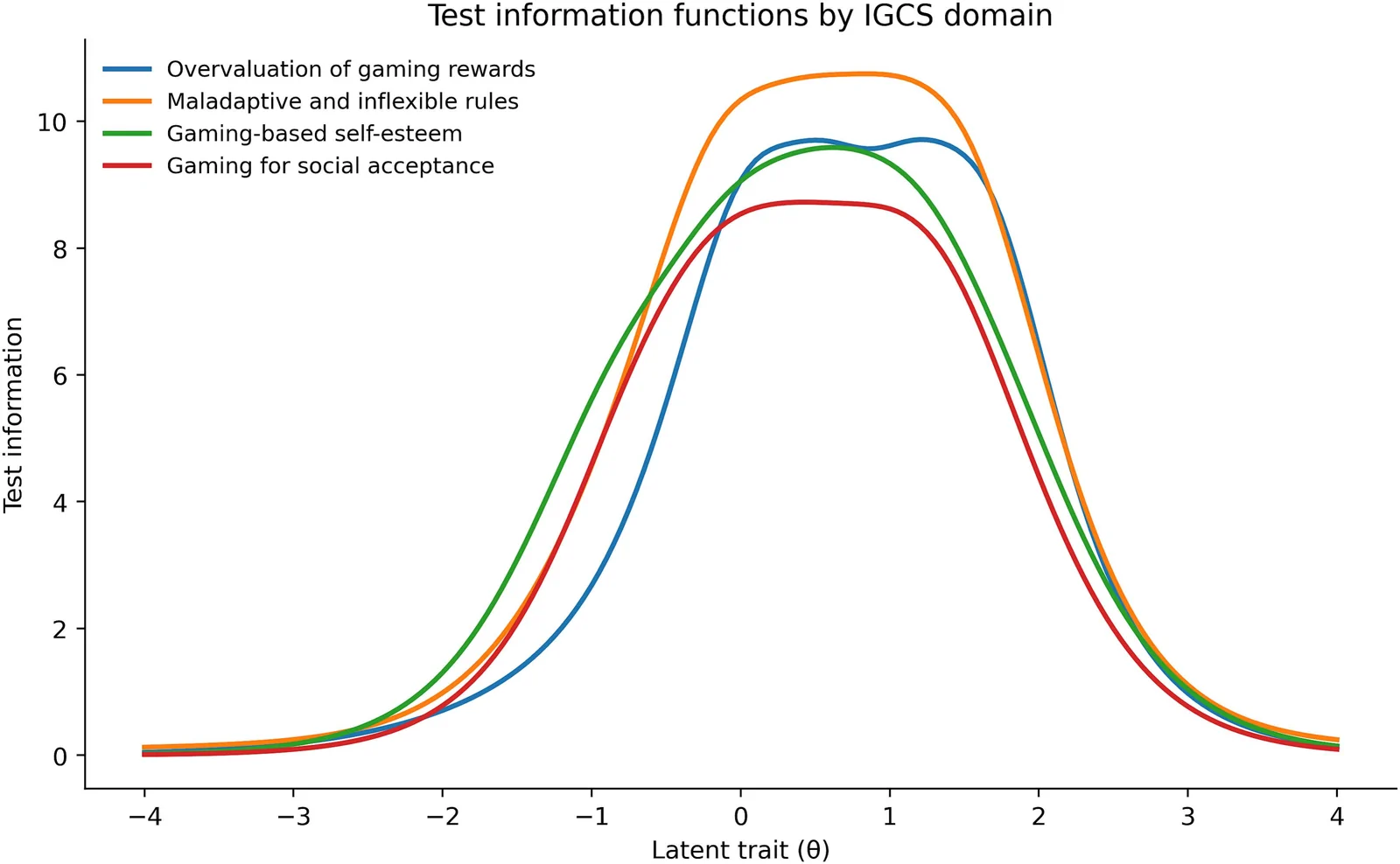
Test information functions for the four IGCS domains under the multidimensional graded response model. Note. Higher information indicates greater measurement precision at the corresponding latent-trait level (θ).

The four-factor GRM fit better than the unidimensional model, Δχ^2^(6) = 212.32, *p* < .001, and had lower AIC and BIC. Absolute fit remained imperfect, M2(246) = 3581.85, CFI = 0.95, TLI = 0.94, RMSEA = 0.11, and SRMSR = 0.08. Latent-domain correlations ranged from 0.89 to 0.96. Discrimination ranged from 0.44 to 3.77; E1 (0.44) and E2 (0.53) were the only values below 1.00. All thresholds were ordered. Empirical reliability ranged from 0.92 to 0.94, and information was greatest from approximately average to moderately elevated trait levels (Fig. 2; Table 3; Supplementary Tables S9–S10).

Mokken H was 0.49 for rewards, 0.36 for rules, 0.53 for gaming-based self-esteem, and 0.55 for social acceptance. Response functions were generally increasing, with minor local reversals, especially in the rules domain. The strongest Q3 residual correlation was E19–E24 (0.66), followed by E13–E14 (0.34). Models excluding E19 or E24 both improved descriptive absolute-fit indices, with negligible differences slightly favoring removal of E24. The evidence identified candidates for future review but did not justify changing the original scale (Supplementary Table S11).

### 3.5 External associations

The total score correlated positively with gaming-problem severity, *r* = 0.66, *p* < .001, and negatively with self-esteem, *r* = −0.24, *p* < .001. All four domains showed the same pattern: correlations with gaming problems ranged from 0.56 to 0.64, and correlations with self-esteem from −0.15 to −0.25 (Table 3; Supplementary Table S12).

Restricting the sample to respondents aged 12–18 years produced essentially unchanged score-level findings. Total-score reliability remained α = 0.94, the correlation with gaming-problem severity was *r* = 0.67, and the correlation with self-esteem was *r* = −0.23. The inter-item correlation pattern was also highly stable, with a mean absolute change of 0.004 across item correlations.

## 4 Discussion

This study provided only partial support for the original IGCS structure. The correlated four-factor CFA showed strong incremental but borderline absolute fit, and the multidimensional GRM outperformed a unidimensional model. However, very high factor correlations, AVE-shared variance failures, and HTMT values approaching unity showed that the four domains were not clearly distinct. Neither the inadmissible bifactor model nor the constrained, poorer-fitting second-order model established a defensible general-factor representation. Thus, the findings do not demonstrate strict unidimensionality, but they also do not support treating the domains as independent cognitive profiles.

This mixed pattern is consistent with the non-uniform cross-cultural literature on the IGCS. The original adolescent study supported four theoretically derived domains, whereas the Chinese adolescent adaptation required a shorter three-factor solution and the Turkish adaptation, conducted in a mixed adolescent-adult sample, retained a multidimensional specification (Cakiroglu & Gormez, 2020; King & Delfabbro, 2016; Yu et al., 2020). The present Persian findings therefore resemble the original and Turkish studies in showing relative support for a multidimensional representation, but they also reveal substantial empirical overlap among the domains. In this respect, the results caution against assuming that retention of a four-factor specification necessarily implies that the four domain scores function as clearly distinct constructs.

Differences across studies may reflect language, item content, sample age, cultural meanings attached to gaming reward and competence, and the extent to which gaming contributes to self-worth and peer recognition. Importantly, the original and Chinese studies both focused on adolescents but yielded different structures, suggesting that developmental stage alone cannot account for the observed variation. The current findings extend this international evidence by showing that, in a Persian-speaking adolescent-focused sample, the original domains remain comparatively useful for organizing item content, yet are more difficult to distinguish empirically than their theoretical definitions might suggest. Cross-cultural replication should therefore evaluate both overall model fit and the distinctiveness of the proposed cognitive domains rather than relying solely on whether the nominal factor structure can be reproduced.

E1 and E2 were the clearest item-level weaknesses: both had low CFA loadings, weak corrected correlations, and discrimination below 1.00. Their wording may depend strongly on game genre or experience, but that explanation remains tentative without cognitive interviewing. Removing them modestly improved some indices without resolving factor overlap, so the 22-item analysis does not validate a shortened scale. E19 and E24 also shared near-duplicate content concerning recognition and respect from other players. Their pronounced local dependence, and the negligible difference between removal models, supports future rewording or comparative testing rather than immediate deletion.

Ordered thresholds indicated that higher response categories generally represented higher latent-trait levels, but the marked concentration at the lowest category and reduced information at trait extremes qualify this conclusion. The Persian IGCS appears most precise around average to moderately elevated cognition levels. The rules domain was comparatively weak in both Mokken scalability and item discrimination, reinforcing the need for item review.

The strong association with gaming-problem severity was consistent with cognitive-behavioral accounts and prior empirical work. The inverse association with self-esteem was smaller, as hypothesized, and supports theoretical relatedness without implying construct equivalence. Because both variables were self-reported and measured concurrently, these associations do not establish causality, diagnostic accuracy, or criterion validity. The older gaming questionnaire also limits comparability with current DSM-5 and ICD-11 symptom measures.

Gender invariance was not established because the configural model fit poorly. Without an adequately comparable baseline structure, latent mean differences between girls and boys cannot be interpreted confidently: an observed difference could reflect non-equivalent measurement rather than a true difference in gaming-related cognitions. Future research should examine item-level differential functioning and determine whether particular items are understood differently across gender groups.

A major strength of this study is the use of complementary methods that answer different psychometric questions. CFA and factor-distinctiveness indices addressed score structure, multidimensional IRT and Mokken analyses examined item performance, and sensitivity analyses clarified whether the main conclusions depended on a small number of problematic items. For research use, the total score is the most defensible broad summary in this sample. Domain scores may still be useful for theory-driven questions, but their overlap should be reported explicitly and they should not be treated as independent cognitive profiles. The present data do not support diagnostic or screening use.

## 5 Limitations

Several limitations should be considered. Convenience-based online recruitment limits representativeness, and detailed information on geographic distribution, school type, and grade was not consistently available. Although 98.2% of respondents reported ages within the intended 12–18-year range, 1.8% reported values outside that range. Age-restricted sensitivity analyses produced essentially unchanged score-level findings, reducing concern that these observations materially influenced the reported associations and reliability estimates. The cross-sectional, self-report design may have increased shared-method variance and cannot establish temporal or causal relationships. No diagnostic interview was administered. The gaming-problem measure predates current diagnostic frameworks, and the factor structures of the two external measures were not independently tested in this sample, although both showed acceptable-to-high internal consistency. Gender invariance was not supported, but item-level differential functioning was not examined. Finally, the item-removal analyses were post hoc and conducted in the same sample. Independent replication, cognitive interviewing, test-retest assessment, and validation against contemporary clinical criteria are therefore needed.

## 6 Conclusion

The Persian IGCS showed strong reliability and a robust association with gaming problems, but its four domains overlapped substantially. A correlated four-factor representation was relatively superior to a unidimensional model, while E1 and E2 performed weakly and E19 and E24 showed marked local dependence. The total score may be used as a broad research summary, whereas domain scores require cautious, theory-led interpretation. Independent validation and targeted item review are needed before screening, diagnosis, or individual clinical use.

## Declaration of generative AI and AI-assisted technologies in the manuscript preparation process

During the preparation of this work, the authors used ChatGPT (OpenAI) to assist with language editing, readability, and organization. The authors subsequently reviewed and edited the material, verified all statistical results and scientific interpretations, and take full responsibility for the content of the article.

## CRediT authorship contribution statement

**Fereshteh Rajaeiramsheh:** Writing – original draft, Investigation, Data curation, Conceptualization. **Hojjatollah Farahani:** Software, Methodology, Formal analysis. **Ahmad Ashouri:** Writing – review & editing, Supervision. **Komeil Zahedi Tajrishi:** Writing – review & editing, Supervision.

## Ethics approval and informed consent

The study was approved by the Ethics Committee of Iran University of Medical Sciences (IR.IUMS.REC.1404.06). Electronic informed consent was obtained from a parent or legal guardian for every participant. Respondents younger than 18 years additionally provided assent, whereas those reporting age 18 or older provided their own informed consent in addition to parental consent.

## Funding

This research received no external funding.

## Declaration of competing interest

The authors declare that they have no known competing financial interests or personal relationships that could have appeared to influence the work reported in this paper.

## Data Availability

The data supporting the findings of this study are not publicly available because they contain confidential information from adolescent participants. De-identified data may be made available by the corresponding author upon reasonable request, subject to applicable ethical and institutional requirements.
