# Supplementary material for: The Persian internet gaming cognition scale: A psychometric evaluation among Iranian adolescents
Source: Addict Behav Rep. 2026 Aug 28;24:100745. doi: 10.1016/j.abrep.2026.100745 (PMC13560020; doi:10.1016/j.abrep.2026.100745)
Supplement: Supplementary file 1 [file mmc1.docx]

**Supplementary Material**

Manuscript number: ABREP-D-26-00202

***The Persian Internet Gaming Cognition Scale: A psychometric evaluation among Iranian adolescents***

*Detailed item-level estimates are reported to three decimal places to preserve psychometric precision; narrative results in the main manuscript are rounded to two decimal places.*

**Table S1**

*Original English and Persian-translated IGCS items*

| **Domain** | **Item** | **Original English item** | **Persian translation** |
| --- | --- | --- | --- |
| Overvaluation of gaming rewards | E3 | Rewards in Internet games are as important to me as anything else in my life. | پاداش‌هایی که در بازی‌های اینترنتی دریافت می‌کنم، به اندازه سایر جنبه‌های زندگی‌ام برایم مهم هستند. |
| Overvaluation of gaming rewards | E4 | When my game character achieves something, I feel like I have achieved that too. | وقتی کاراکتر (قهرمان) من در بازی به موفقیتی می‌رسد، احساس می‌کنم خودم هم به آن موفقیت دست یافته‌ام. |
| Overvaluation of gaming rewards | E6 | Playing Internet games has many other benefits in my life. | بازی‌های اینترنتی مزایای زیادی در زندگی من دارند. |
| Overvaluation of gaming rewards | E13 | I find myself thinking about video games when I am not playing. | حتی زمانی که در حال بازی کردن نیستم، به بازی‌های ویدیویی فکر می‌کنم. |
| Overvaluation of gaming rewards | E14 | I spend time planning or thinking about the next thing I need to do in a game. | زمانی را صرف برنامه‌ریزی یا تفکر درباره کاری می‌کنم که لازم است در ادامه بازی انجام دهم. |
| Maladaptive and inflexible rules about gaming | E1 | When I make mistakes, lose progress, or fail in a game, I must reload and try again. | وقتی در بازی اشتباهی مرتکب شوم، پیشرفتم کم شود یا شکست بخورم، باید مجدداً از ابتدا آغاز کنم و دوباره تلاش کنم. |
| Maladaptive and inflexible rules about gaming | E2 | It would be a waste to stop playing a game I have already invested so much time and energy in. | کنار گذاشتن بازی‌ای که وقت و انرژی زیادی در آن سرمایه‌گذاری کرده‌ام، اتلاف وقت است. |
| Maladaptive and inflexible rules about gaming | E8 | When I have a goal or objective in a video game, I must complete it as soon as possible. | وقتی در یک بازی ویدیویی مقصود یا هدفی دارم، باید هرچه سریع‌تر به آن برسم. |
| Maladaptive and inflexible rules about gaming | E9 | I prioritize video games before doing something else, e.g., homework or chores. | بازی‌های ویدیویی را به وظایف دیگری مانند تکالیف درسی یا کارهای خانه ترجیح می‌دهم. |
| Maladaptive and inflexible rules about gaming | E10 | I tell myself “just a few more minutes” when I play a game, but then play much longer. | هنگام بازی به خودم می‌گویم «فقط چند دقیقه دیگر»، اما بسیار بیشتر از آن بازی می‌کنم. |
| Maladaptive and inflexible rules about gaming | E11 | I feel uncomfortable thinking about my unfinished goals or objectives in video games. | من از فکر کردن به اهداف یا مأموریت‌های ناتمام در بازی‌های ویدیویی احساس ناراحتی می‌کنم. |
| Maladaptive and inflexible rules about gaming | E15 | I feel unsatisfied until I have done everything I want to do in a video game. | تا زمانی که تمام کارهایی را که می‌خواهم در یک بازی ویدیویی انجام دهم به پایان نرسانده‌ام، خیالم راحت نیست. |
| Maladaptive and inflexible rules about gaming | E16 | No amount of gaming time ever feels like “long enough”. | هیچ‌وقت میزان زمانی که صرف بازی می‌کنم، به اندازه کافی طولانی احساس نمی‌شود. |
| Gaming-based self-esteem | E5 | I tend to feel better after playing Internet games. | بعد از انجام بازی‌های اینترنتی احساس بهتری دارم. |
| Gaming-based self-esteem | E7 | I would feel bad if I was not able to play Internet games. | اگر نتوانم بازی‌های اینترنتی انجام دهم، احساس بدی خواهم داشت. |
| Gaming-based self-esteem | E12 | I am proud of my gaming achievements. | من به دستاوردهایی که در بازی به دست می‌آورم، افتخار می‌کنم. |
| Gaming-based self-esteem | E20 | I feel more in control when I play video games. | هنگام انجام بازی‌های ویدیویی، کنترل بیشتری بر خودم دارم. |
| Gaming-based self-esteem | E21 | I would not be able to cope with stress in my life without video games. | بدون انجام بازی‌های ویدیویی نمی‌توانم با استرس در زندگی‌ام مقابله کنم. |
| Gaming-based self-esteem | E23 | If I complete or master an achievement, skill or goal in a video game, I feel good about myself. | اگر در یک بازی ویدیویی به موفقیت، مهارت یا هدفی دست یابم یا بر آن مسلط شوم، نسبت به خودم احساس خوبی دارم. |
| Gaming as a means of social acceptance | E17 | People who do not play video games do not really understand an important part of who I am. | افرادی که بازی‌های ویدیویی انجام نمی‌دهند، واقعاً بخش مهمی از هویت من را درک نمی‌کنند. |
| Gaming as a means of social acceptance | E18 | It is important to me that I am better at certain video games than other players. | برایم مهم است که در برخی بازی‌های ویدیویی از بازیکنان دیگر بهتر باشم. |
| Gaming as a means of social acceptance | E19 | Other players admire and respect my gaming achievements. | بازیکنان دیگر موفقیت‌های مرا در بازی تحسین می‌کنند و به آن احترام می‌گذارند. |
| Gaming as a means of social acceptance | E22 | I feel safer and more comfortable playing a video game than in most other social situations. | هنگام بازی، در مقایسه با بیشتر موقعیت‌های اجتماعی دیگر، احساس امنیت و راحتی بیشتری دارم. |
| Gaming as a means of social acceptance | E24 | When I succeed in a video game, players notice and respect me. | وقتی در یک بازی ویدیویی موفق می‌شوم، بازیکنان متوجه موفقیتم می‌شوند و به من احترام می‌گذارند. |

*Note.* The Persian version retained the 24 items, four-domain organization, response options, and scoring procedure of the original IGCS. The original English items are reproduced with permission from the scale author.

**Table S2**

*Item-level descriptive statistics and response-endpoint frequencies for the Persian IGCS (N = 1,191)*

| **Item** | **Mean** | **SD** | **Median** | **Min** | **Max** | **P25** | **P75** | **Skewness** | **Kurtosis** | **Floor % (0)** | **Ceiling % (4)** |
| --- | --- | --- | --- | --- | --- | --- | --- | --- | --- | --- | --- |
| E1 | 2.133 | 1.387 | 2 | 0 | 4 | 1 | 3 | -0.136 | 1.815 | 17.63 | 22.17 |
| E2 | 1.664 | 1.315 | 2 | 0 | 4 | 1 | 3 | 0.305 | 1.994 | 24.43 | 11.92 |
| E3 | 1.307 | 1.356 | 1 | 0 | 4 | 0 | 2 | 0.656 | 2.172 | 40.22 | 9.82 |
| E4 | 2.027 | 1.420 | 2 | 0 | 4 | 1 | 3 | -0.021 | 1.725 | 20.15 | 21.41 |
| E5 | 1.792 | 1.434 | 2 | 0 | 4 | 0 | 3 | 0.199 | 1.727 | 26.03 | 17.72 |
| E6 | 1.311 | 1.342 | 1 | 0 | 4 | 0 | 2 | 0.701 | 2.285 | 38.12 | 10.33 |
| E7 | 1.023 | 1.236 | 1 | 0 | 4 | 0 | 2 | 0.985 | 2.885 | 49.12 | 6.21 |
| E8 | 1.667 | 1.360 | 2 | 0 | 4 | 0 | 3 | 0.311 | 1.892 | 26.03 | 13.18 |
| E9 | 1.236 | 1.354 | 1 | 0 | 4 | 0 | 2 | 0.738 | 2.287 | 43.83 | 9.66 |
| E10 | 1.542 | 1.379 | 1 | 0 | 4 | 0 | 3 | 0.423 | 1.946 | 31.65 | 12.68 |
| E11 | 1.181 | 1.278 | 1 | 0 | 4 | 0 | 2 | 0.826 | 2.575 | 41.48 | 7.89 |
| E12 | 1.920 | 1.431 | 2 | 0 | 4 | 1 | 3 | 0.094 | 1.707 | 22.33 | 20.07 |
| E13 | 1.024 | 1.241 | 1 | 0 | 4 | 0 | 2 | 0.981 | 2.850 | 49.29 | 6.13 |
| E14 | 1.156 | 1.225 | 1 | 0 | 4 | 0 | 2 | 0.766 | 2.549 | 41.56 | 5.79 |
| E15 | 1.226 | 1.336 | 1 | 0 | 4 | 0 | 2 | 0.756 | 2.328 | 42.82 | 8.98 |
| E16 | 1.676 | 1.420 | 2 | 0 | 4 | 0 | 3 | 0.261 | 1.769 | 30.06 | 14.69 |
| E17 | 1.298 | 1.403 | 1 | 0 | 4 | 0 | 2 | 0.702 | 2.167 | 42.65 | 11.84 |
| E18 | 1.792 | 1.453 | 2 | 0 | 4 | 0 | 3 | 0.156 | 1.647 | 27.54 | 16.88 |
| E19 | 1.573 | 1.344 | 1 | 0 | 4 | 0 | 2 | 0.395 | 2.005 | 28.88 | 12.09 |
| E20 | 1.754 | 1.428 | 2 | 0 | 4 | 0 | 3 | 0.218 | 1.745 | 27.29 | 16.62 |
| E21 | 0.915 | 1.244 | 0 | 0 | 4 | 0 | 2 | 1.165 | 3.164 | 55.75 | 6.05 |
| E22 | 1.196 | 1.293 | 1 | 0 | 4 | 0 | 2 | 0.783 | 2.474 | 42.23 | 8.06 |
| E23 | 1.902 | 1.400 | 2 | 0 | 4 | 1 | 3 | 0.094 | 1.770 | 22.00 | 18.30 |
| E24 | 1.597 | 1.342 | 1 | 0 | 4 | 0 | 2 | 0.384 | 2.015 | 27.79 | 12.59 |

*Note.* Floor and ceiling percentages are the proportions selecting the lowest (0) and highest (4) response categories, respectively. A 15% threshold was prespecified for identifying potential floor or ceiling effects. No item-level responses were missing.

**Table S3**

*Complete confirmatory factor-analysis loadings for the original 24-item Persian IGCS (DWLS; N = 1,191)*

| **Factor** | **Item** | **Estimate (b)** | **SE** | **Z** | **p** | **Std. loading (Std.all)** |
| --- | --- | --- | --- | --- | --- | --- |
| F2 | E1 | 1.000 |  |  |  | 0.277 |
| F2 | E2 | 0.914 | 0.042 | 21.785 | < .001 | 0.254 |
| F1 | E3 | 1.000 |  |  |  | 0.731 |
| F1 | E4 | 0.951 | 0.014 | 70.183 | < .001 | 0.695 |
| F3 | E5 | 1.000 |  |  |  | 0.799 |
| F1 | E6 | 1.023 | 0.014 | 75.566 | < .001 | 0.747 |
| F3 | E7 | 1.009 | 0.011 | 90.846 | < .001 | 0.806 |
| F2 | E8 | 2.712 | 0.088 | 30.815 | < .001 | 0.752 |
| F2 | E9 | 2.678 | 0.087 | 30.762 | < .001 | 0.743 |
| F2 | E10 | 2.547 | 0.083 | 30.599 | < .001 | 0.707 |
| F2 | E11 | 2.867 | 0.092 | 31.030 | < .001 | 0.795 |
| F3 | E12 | 1.007 | 0.011 | 93.197 | < .001 | 0.804 |
| F1 | E13 | 1.227 | 0.014 | 84.891 | < .001 | 0.896 |
| F1 | E14 | 1.157 | 0.014 | 83.516 | < .001 | 0.845 |
| F2 | E15 | 3.030 | 0.097 | 31.189 | < .001 | 0.841 |
| F2 | E16 | 2.749 | 0.089 | 30.904 | < .001 | 0.763 |
| F4 | E17 | 1.000 |  |  |  | 0.826 |
| F4 | E18 | 0.992 | 0.011 | 90.955 | < .001 | 0.819 |
| F4 | E19 | 0.798 | 0.011 | 74.260 | < .001 | 0.659 |
| F3 | E20 | 0.891 | 0.011 | 83.647 | < .001 | 0.711 |
| F3 | E21 | 0.901 | 0.011 | 80.409 | < .001 | 0.720 |
| F4 | E22 | 0.939 | 0.011 | 86.339 | < .001 | 0.776 |
| F3 | E23 | 0.920 | 0.011 | 86.557 | < .001 | 0.735 |
| F4 | E24 | 0.798 | 0.011 | 74.516 | < .001 | 0.659 |

*Note.* DWLS = diagonally weighted least squares. Blank standard errors and z values identify marker indicators fixed for scale identification.

**Table S4**

*Item-total and item-subscale statistics for the Persian IGCS*

| **Factor** | **Item** | **Corrected item–total r** | **Total α if deleted** | **Corrected item–subscale r** | **Subscale α if deleted** |
| --- | --- | --- | --- | --- | --- |
| F2 | E1 | 0.249 | 0.947 | 0.237 | 0.825 |
| F2 | E2 | 0.226 | 0.947 | 0.247 | 0.822 |
| F1 | E3 | 0.632 | 0.942 | 0.621 | 0.760 |
| F1 | E4 | 0.599 | 0.942 | 0.482 | 0.805 |
| F3 | E5 | 0.725 | 0.941 | 0.720 | 0.808 |
| F1 | E6 | 0.623 | 0.942 | 0.543 | 0.784 |
| F3 | E7 | 0.696 | 0.941 | 0.644 | 0.824 |
| F2 | E8 | 0.673 | 0.941 | 0.599 | 0.772 |
| F2 | E9 | 0.620 | 0.942 | 0.591 | 0.773 |
| F2 | E10 | 0.608 | 0.942 | 0.624 | 0.768 |
| F2 | E11 | 0.678 | 0.941 | 0.654 | 0.765 |
| F3 | E12 | 0.729 | 0.941 | 0.645 | 0.823 |
| F1 | E13 | 0.709 | 0.941 | 0.682 | 0.743 |
| F1 | E14 | 0.687 | 0.941 | 0.653 | 0.752 |
| F2 | E15 | 0.732 | 0.941 | 0.667 | 0.762 |
| F2 | E16 | 0.663 | 0.942 | 0.575 | 0.775 |
| F4 | E17 | 0.711 | 0.941 | 0.611 | 0.823 |
| F4 | E18 | 0.723 | 0.941 | 0.685 | 0.802 |
| F4 | E19 | 0.601 | 0.942 | 0.700 | 0.798 |
| F3 | E20 | 0.632 | 0.942 | 0.627 | 0.827 |
| F3 | E21 | 0.594 | 0.942 | 0.561 | 0.838 |
| F4 | E22 | 0.673 | 0.941 | 0.555 | 0.836 |
| F3 | E23 | 0.656 | 0.942 | 0.612 | 0.830 |
| F4 | E24 | 0.594 | 0.942 | 0.702 | 0.798 |

*Note.* Corrected item-total correlations were computed against the 24-item total score excluding the target item. Corrected item-subscale correlations and alpha-if-deleted values were computed within the item’s theoretical domain.

**Table S5**

*Average variance extracted and shared-variance indices*

| **Construct** | **AVE** | **MSV** | **ASV** | **AVE>MSV** | **AVE>ASV** |
| --- | --- | --- | --- | --- | --- |
| F1 | 0.618 | 0.899 | 0.838 | No | No |
| F2 | 0.460 | 0.908 | 0.870 | No | No |
| F3 | 0.583 | 0.978 | 0.929 | No | No |
| F4 | 0.564 | 0.978 | 0.865 | No | No |

*Note.* AVE = average variance extracted; MSV = maximum shared variance; ASV = average shared variance. AVE was below both MSV and ASV for all domains.

**Table S6**

*Heterotrait–monotrait ratios among the four IGCS domains*

| **Construct** | **F1** | **F2** | **F3** | **F4** |
| --- | --- | --- | --- | --- |
| F1 | — | 0.974 | 0.999 | 0.894 |
| F2 | 0.974 | — | 0.948 | 0.900 |
| F3 | 0.999 | 0.948 | — | 0.947 |
| F4 | 0.894 | 0.900 | 0.947 | — |

Note. F1 = overvaluation of gaming rewards; F2 = maladaptive and inflexible rules about gaming; F3 = gaming-based self-esteem; F4 = gaming as a means of social acceptance.

**Table S7**

*Gender measurement-invariance model fit*

| **Model** | **χ²** | **df** | **CFI** | **TLI** | **RMSEA** | **SRMR** | **ΔCFI** | **ΔRMSEA** |
| --- | --- | --- | --- | --- | --- | --- | --- | --- |
| Configural | 4883.76 | 492 | .896 | .883 | .123 | .084 | — | — |
| Metric (loadings equal) | 3735.56 | 512 | .924 | .918 | .103 | .088 | .028 | -.020 |
| Scalar (loadings and thresholds equal) | 4631.89 | 560 | .903 | .905 | .111 | .084 | -.020 | .008 |

*Note.* The configural model showed inadequate fit; therefore, measurement invariance was not established and subsequent group comparisons should not be interpreted as invariant-score comparisons.

**Table S8**

*Exploratory latent-mean estimates for group 2 relative to group 1*

| **Factor** | **Group 2 estimate** | **SE** | **z** | **p** | **95% CI** |
| --- | --- | --- | --- | --- | --- |
| F1 | -.572 | .069 | -8.23 | < .001 | [-0.708, -0.436] |
| F2 | -.206 | .031 | -6.60 | < .001 | [-0.267, -0.145] |
| F3 | -.851 | .079 | -10.73 | < .001 | [-1.006, -0.695] |
| F4 | -.729 | .096 | -7.60 | < .001 | [-0.917, -0.541] |

*Note.* Group 1 was fixed at 0, and group 2 estimates represent differences from group 1. These estimates are presented for transparency but should not be substantively interpreted because configural invariance was not supported.

**Table S9**

*Multidimensional graded response model item parameters*

| **Item** | **a1** | **a2** | **a3** | **a4** | **b1** | **b2** | **b3** | **b4** |
| --- | --- | --- | --- | --- | --- | --- | --- | --- |
| E1 | 0.000 | 0.437 | 0.000 | 0.000 | -3.603 | -1.623 | 0.879 | 3.043 |
| E2 | 0.000 | 0.529 | 0.000 | 0.000 | -2.205 | -0.078 | 2.067 | 4.017 |
| E3 | 1.856 | 0.000 | 0.000 | 0.000 | -0.266 | 0.410 | 1.110 | 1.763 |
| E4 | 1.438 | 0.000 | 0.000 | 0.000 | -1.259 | -0.407 | 0.479 | 1.244 |
| E5 | 0.000 | 0.000 | 2.542 | 0.000 | -0.704 | -0.044 | 0.591 | 1.127 |
| E6 | 1.957 | 0.000 | 0.000 | 0.000 | -0.329 | 0.454 | 1.120 | 1.672 |
| E7 | 0.000 | 0.000 | 2.685 | 0.000 | 0.063 | 0.611 | 1.261 | 1.769 |
| E8 | 0.000 | 1.954 | 0.000 | 0.000 | -0.789 | 0.070 | 0.786 | 1.480 |
| E9 | 0.000 | 2.174 | 0.000 | 0.000 | -0.099 | 0.462 | 1.133 | 1.657 |
| E10 | 0.000 | 1.941 | 0.000 | 0.000 | -0.555 | 0.161 | 0.899 | 1.516 |
| E11 | 0.000 | 2.693 | 0.000 | 0.000 | -0.159 | 0.529 | 1.134 | 1.630 |
| E12 | 0.000 | 0.000 | 2.416 | 0.000 | -0.874 | -0.140 | 0.531 | 1.065 |
| E13 | 3.765 | 0.000 | 0.000 | 0.000 | 0.071 | 0.590 | 1.157 | 1.619 |
| E14 | 2.965 | 0.000 | 0.000 | 0.000 | -0.150 | 0.479 | 1.191 | 1.780 |
| E15 | 0.000 | 3.169 | 0.000 | 0.000 | -0.108 | 0.447 | 0.969 | 1.470 |
| E16 | 0.000 | 2.175 | 0.000 | 0.000 | -0.570 | 0.037 | 0.730 | 1.338 |
| E17 | 0.000 | 0.000 | 0.000 | 2.790 | -0.122 | 0.387 | 0.935 | 1.364 |
| E18 | 0.000 | 0.000 | 0.000 | 2.724 | -0.634 | -0.014 | 0.510 | 1.138 |
| E19 | 0.000 | 0.000 | 0.000 | 2.061 | -0.645 | 0.115 | 0.913 | 1.518 |
| E20 | 0.000 | 0.000 | 1.969 | 0.000 | -0.720 | -0.016 | 0.689 | 1.292 |
| E21 | 0.000 | 0.000 | 1.954 | 0.000 | 0.259 | 0.838 | 1.436 | 2.058 |
| E22 | 0.000 | 0.000 | 0.000 | 2.154 | -0.177 | 0.488 | 1.218 | 1.774 |
| E23 | 0.000 | 0.000 | 1.957 | 0.000 | -0.977 | -0.212 | 0.566 | 1.203 |
| E24 | 0.000 | 0.000 | 0.000 | 2.052 | -0.690 | 0.101 | 0.922 | 1.486 |

*Note.* a1–a4 are discrimination parameters for F1–F4, respectively; non-target discrimination parameters were fixed at 0. b1–b4 are ordered category thresholds on the latent-trait scale.

**Table S10**

*Factor correlations, Mokken scalability, and empirical reliability in the multidimensional GRM*

***Panel A. Estimated factor correlations***

| **Factor** | **F1** | **F2** | **F3** | **F4** |
| --- | --- | --- | --- | --- |
| F1 | 1.000 | 0.962 | 0.962 | 0.886 |
| F2 | 0.962 | 1.000 | 0.959 | 0.911 |
| F3 | 0.962 | 0.959 | 1.000 | 0.949 |
| F4 | 0.886 | 0.911 | 0.949 | 1.000 |

***Panel B. Domain-level scalability and empirical reliability***

| **Factor** | **Domain** | **Mokken H (SE)** | **EAP reliability** |
| --- | --- | --- | --- |
| F1 | Overvaluation of gaming rewards | .485 (.017) | .934 |
| F2 | Maladaptive and inflexible rules about gaming | .363 (.014) | .937 |
| F3 | Gaming-based self-esteem | .527 (.015) | .941 |
| F4 | Gaming as a means of social acceptance | .546 (.016) | .918 |

*Note.* H = Mokken scalability coefficient; EAP = expected a posteriori. The machine-readable monotonicity summary did not contain item-level violation counts; monotonicity was therefore evaluated from the supplied item response function plots rather than reported numerically.

**Table S11**

*Local dependence and item-removal sensitivity analyses*

***Panel A. Largest positive Yen’s Q3 residual correlations***

| **Item pair** | **Q3** |
| --- | --- |
| E19–E24 | 0.657 |
| E13–E14 | 0.337 |
| E21–E22 | 0.308 |
| E1–E4 | 0.262 |
| E4–E8 | 0.245 |
| E3–E4 | 0.224 |
| E5–E6 | 0.221 |
| E1–E2 | 0.215 |
| E1–E8 | 0.214 |
| E8–E23 | 0.202 |

***Panel B. Models excluding E19 or E24***

| **Model** | **AIC** | **BIC** | **M2 (df)** | **RMSEA** | **SRMSR** | **CFI** | **TLI** |
| --- | --- | --- | --- | --- | --- | --- | --- |
| Full 24-item model | 71,561.87 | 72,202.27 | 3,581.85 (246) | .107 | .082 | .947 | .940 |
| 23-item model excluding E19 | 68,540.78 | 69,155.77 | 2,730.39 (224) | .097 | .073 | .957 | .951 |
| 23-item model excluding E24 | 68,540.13 | 69,155.12 | 2,720.31 (224) | .097 | .072 | .957 | .951 |

***Panel C. 24-item and 22-item sensitivity models***

| **Model** | **AIC** | **BIC** | **M2** | **RMSEA** | **CFI** |
| --- | --- | --- | --- | --- | --- |
| 24-item (full) | 71,561.87 | 72,202.27 | 3,581.85 | .107 | .947 |
| 22-item (drop E1, E2) | 64,145.29 | 64,734.87 | 3,172.04 | .111 | .951 |

*Note.* Q3 values are residual correlations. Model-fit indices for models containing different item sets are descriptive and should not be treated as formal evidence that one scale length is superior. The 22-item model excluded E1 and E2 and was evaluated as a sensitivity analysis only.

**Table S12**

*Correlations among IGCS scores and external measures (N = 1,191)*

| **Variable** | **M** | **SD** | **1** | **2** | **3** | **4** | **5** | **6** | **7** |
| --- | --- | --- | --- | --- | --- | --- | --- | --- | --- |
| 1. IGCS total | 35.91 | 21.50 | — | .908 | .916 | .933 | .887 | .660 | −.237 |
| 2. F1: Overvaluation | 6.82 | 4.95 | .908 | — | .785 | .827 | .731 | .586 | −.226 |
| 3. F2: Maladaptive and inflexible rules | 12.32 | 7.06 | .916 | .785 | — | .780 | .732 | .610 | −.246 |
| 4. F3: Gaming-based self-esteem | 9.31 | 6.19 | .933 | .827 | .780 | — | .797 | .644 | −.232 |
| 5. F4: Gaming as a means of social acceptance | 7.46 | 5.36 | .887 | .731 | .732 | .797 | — | .559 | −.149 |
| 6. Gaming-problem severity | 40.21 | 17.55 | .660 | .586 | .610 | .644 | .559 | — | −.248 |
| 7. Eysenck self-esteem | 17.48 | 5.72 | −.237 | −.226 | −.246 | −.232 | −.149 | −.248 | — |

Note. M = mean; SD = standard deviation. F1 = overvaluation of gaming rewards; F2 = maladaptive and inflexible rules about gaming; F3 = gaming-based self-esteem; F4 = gaming as a means of social acceptance. Self-esteem items were scored according to the keyed direction (1 for the keyed yes/no response, 0 for the opposite response, and 0.5 for uncertain); higher scores indicate greater self-esteem. All off-diagonal correlations were significant at p < .001.

**Supplementary Figures**

*Item-response-function plots used for the visual assessment of monotonicity*

**Figure S1**

*Mokken monotonicity plot for E3 (F1: overvaluation of gaming rewards)*

Note. The left panel displays item-step response functions and the right panel displays the item response function across rest-score groups. Minor local reversals should be interpreted visually rather than as formal violation counts because the machine-readable summary did not provide complete item-level violation statistics.

**Figure S2**

*Mokken monotonicity plot for E4 (F1: overvaluation of gaming rewards)*

Note. The left panel displays item-step response functions and the right panel displays the item response function across rest-score groups. Minor local reversals should be interpreted visually rather than as formal violation counts because the machine-readable summary did not provide complete item-level violation statistics.

**Figure S3**

*Mokken monotonicity plot for E6 (F1: overvaluation of gaming rewards)*

Note. The left panel displays item-step response functions and the right panel displays the item response function across rest-score groups. Minor local reversals should be interpreted visually rather than as formal violation counts because the machine-readable summary did not provide complete item-level violation statistics.

**Figure S4**

*Mokken monotonicity plot for E13 (F1: overvaluation of gaming rewards)*

Note. The left panel displays item-step response functions and the right panel displays the item response function across rest-score groups. Minor local reversals should be interpreted visually rather than as formal violation counts because the machine-readable summary did not provide complete item-level violation statistics.

**Figure S5**

*Mokken monotonicity plot for E14 (F1: overvaluation of gaming rewards)*

Note. The left panel displays item-step response functions and the right panel displays the item response function across rest-score groups. Minor local reversals should be interpreted visually rather than as formal violation counts because the machine-readable summary did not provide complete item-level violation statistics.

**Figure S6**

*Mokken monotonicity plot for E1 (F2: maladaptive and inflexible rules about gaming)*

Note. The left panel displays item-step response functions and the right panel displays the item response function across rest-score groups. Minor local reversals should be interpreted visually rather than as formal violation counts because the machine-readable summary did not provide complete item-level violation statistics.

**Figure S7**

*Mokken monotonicity plot for E2 (F2: maladaptive and inflexible rules about gaming)*

Note. The left panel displays item-step response functions and the right panel displays the item response function across rest-score groups. Minor local reversals should be interpreted visually rather than as formal violation counts because the machine-readable summary did not provide complete item-level violation statistics.

**Figure S8**

*Mokken monotonicity plot for E8 (F2: maladaptive and inflexible rules about gaming)*

Note. The left panel displays item-step response functions and the right panel displays the item response function across rest-score groups. Minor local reversals should be interpreted visually rather than as formal violation counts because the machine-readable summary did not provide complete item-level violation statistics.

**Figure S9**

*Mokken monotonicity plot for E9 (F2: maladaptive and inflexible rules about gaming)*

Note. The left panel displays item-step response functions and the right panel displays the item response function across rest-score groups. Minor local reversals should be interpreted visually rather than as formal violation counts because the machine-readable summary did not provide complete item-level violation statistics.

**Figure S10**

*Mokken monotonicity plot for E10 (F2: maladaptive and inflexible rules about gaming)*

Note. The left panel displays item-step response functions and the right panel displays the item response function across rest-score groups. Minor local reversals should be interpreted visually rather than as formal violation counts because the machine-readable summary did not provide complete item-level violation statistics.

**Figure S11**

*Mokken monotonicity plot for E11 (F2: maladaptive and inflexible rules about gaming)*

Note. The left panel displays item-step response functions and the right panel displays the item response function across rest-score groups. Minor local reversals should be interpreted visually rather than as formal violation counts because the machine-readable summary did not provide complete item-level violation statistics.

**Figure S12**

*Mokken monotonicity plot for E15 (F2: maladaptive and inflexible rules about gaming)*

Note. The left panel displays item-step response functions and the right panel displays the item response function across rest-score groups. Minor local reversals should be interpreted visually rather than as formal violation counts because the machine-readable summary did not provide complete item-level violation statistics.

**Figure S13**

*Mokken monotonicity plot for E16 (F2: maladaptive and inflexible rules about gaming)*

Note. The left panel displays item-step response functions and the right panel displays the item response function across rest-score groups. Minor local reversals should be interpreted visually rather than as formal violation counts because the machine-readable summary did not provide complete item-level violation statistics.

**Figure S14**

*Mokken monotonicity plot for E5 (F3: gaming-based self-esteem)*

Note. The left panel displays item-step response functions and the right panel displays the item response function across rest-score groups. Minor local reversals should be interpreted visually rather than as formal violation counts because the machine-readable summary did not provide complete item-level violation statistics.

**Figure S15**

*Mokken monotonicity plot for E7 (F3: gaming-based self-esteem)*

Note. The left panel displays item-step response functions and the right panel displays the item response function across rest-score groups. Minor local reversals should be interpreted visually rather than as formal violation counts because the machine-readable summary did not provide complete item-level violation statistics.

**Figure S16**

*Mokken monotonicity plot for E12 (F3: gaming-based self-esteem)*

Note. The left panel displays item-step response functions and the right panel displays the item response function across rest-score groups. Minor local reversals should be interpreted visually rather than as formal violation counts because the machine-readable summary did not provide complete item-level violation statistics.

**Figure S17**

*Mokken monotonicity plot for E20 (F3: gaming-based self-esteem)*

Note. The left panel displays item-step response functions and the right panel displays the item response function across rest-score groups. Minor local reversals should be interpreted visually rather than as formal violation counts because the machine-readable summary did not provide complete item-level violation statistics.

**Figure S18**

*Mokken monotonicity plot for E21 (F3: gaming-based self-esteem)*

Note. The left panel displays item-step response functions and the right panel displays the item response function across rest-score groups. Minor local reversals should be interpreted visually rather than as formal violation counts because the machine-readable summary did not provide complete item-level violation statistics.

**Figure S19**

*Mokken monotonicity plot for E23 (F3: gaming-based self-esteem)*

Note. The left panel displays item-step response functions and the right panel displays the item response function across rest-score groups. Minor local reversals should be interpreted visually rather than as formal violation counts because the machine-readable summary did not provide complete item-level violation statistics.

**Figure S20**

*Mokken monotonicity plot for E17 (F4: gaming as a means of social acceptance)*

Note. The left panel displays item-step response functions and the right panel displays the item response function across rest-score groups. Minor local reversals should be interpreted visually rather than as formal violation counts because the machine-readable summary did not provide complete item-level violation statistics.

**Figure S21**

*Mokken monotonicity plot for E18 (F4: gaming as a means of social acceptance)*

Note. The left panel displays item-step response functions and the right panel displays the item response function across rest-score groups. Minor local reversals should be interpreted visually rather than as formal violation counts because the machine-readable summary did not provide complete item-level violation statistics.

**Figure S22**

*Mokken monotonicity plot for E19 (F4: gaming as a means of social acceptance)*

Note. The left panel displays item-step response functions and the right panel displays the item response function across rest-score groups. Minor local reversals should be interpreted visually rather than as formal violation counts because the machine-readable summary did not provide complete item-level violation statistics.

**Figure S23**

*Mokken monotonicity plot for E22 (F4: gaming as a means of social acceptance)*

Note. The left panel displays item-step response functions and the right panel displays the item response function across rest-score groups. Minor local reversals should be interpreted visually rather than as formal violation counts because the machine-readable summary did not provide complete item-level violation statistics.

**Figure S24**

*Mokken monotonicity plot for E24 (F4: gaming as a means of social acceptance)*

Note. The left panel displays item-step response functions and the right panel displays the item response function across rest-score groups. Minor local reversals should be interpreted visually rather than as formal violation counts because the machine-readable summary did not provide complete item-level violation statistics.
